# Macrocognition in Day-To-Day Police Incident Response

**DOI:** 10.3389/fpsyg.2016.00293

**Published:** 2016-03-08

**Authors:** Chris Baber, Richard McMaster

**Affiliations:** School of Engineering, University of BirminghamBirmingham, UK

**Keywords:** macrocognition, sensemaking, police, incident response

## Abstract

Using examples of incidents that UK Police Forces deal with on a day-to-day basis, we explore the macrocognition of incident response. Central to our analysis is the idea that information relating to an incident is translated from negotiated to structured and actionable meaning, in terms of the Community of Practice of the personnel involved in incident response. Through participant observation of, and interviews with, police personnel, we explore the manner in which these different types of meaning shift over the course of incident. In this way, macrocognition relates to gathering, framing, and sharing information through the collaborative sensemaking practices of those involved. This involves two cycles of macrocognition, which we see as ‘informal’ (driven by information gathering as the Community of Practice negotiates and actions meaning) and ‘formal’ (driven by the need to assign resources to the response and the need to record incident details). The examples illustrate that these cycles are often intertwined, as are the different forms of meaning, in situation-specific ways that provide adaptive response to the demands of the incident.

## Introduction

We consider Police incident response as a form of macrocognition (Klein et al., 2003). The primary research question relates to the manner in which a collection of individuals, a ‘community of practice’ (Wenger et al., 2002), develop a common understanding of the problem that they are addressing through processes of sensemaking. We propose that sensemaking can be a collaborative activity within a given community of practice. This activity is shaped by the institutional frames of the community of practice, which define the formal and informal rules by which information is defined and shared. These rules can be seen in the manner in which the community of practice manages ‘meaning’, in its collaborative sensemaking. We consider three types of meaning, which we term ‘negotiated’ (in which informal, unstructured accounts of the incident are shared and clarified), ‘structured’ (in which formal accounts are logged), and ‘actionable’ (in which the commentary on the incident informs decisions on how to resource and manage the response). For this paper, a key issue in macrocognition, therefore, relates to this question of how these different meanings are recognized and managed.

In terms of ‘community of practice’, the incidents that we consider involve Standard Operating Procedures. This means that there is an established organization of individuals, operating within a well-defined domain, and who “…*share a common set of patterns of interpretation, implicit assumptions, and beliefs…*” (Burnett et al., 2004, p. 12). The manner in which a Community of Practice shares its knowledge and understanding involves what we have previously called Collaborative Sensemaking (Duffy and Baber, 2013), which combines ‘semantic’ sensemaking (in which a group of people seek to develop a common interpretation of an event, i.e., determining what is known) and ‘pragmatic’ sensemaking (in which a group of people can be allocated different roles in terms of holding or sharing information, i.e., determining who knows what).

From the point of view of ‘pragmatic sensemaking’, a Community of Practice shares information partly through common jargon (and associated experience and ‘world view’) and partly through shared communication technologies and practices. An irony of this (for the type of incident response considered in this paper) is that ‘outsiders’ (i.e., people who are not part of the Community of Practice) are the very focus of its activity. One implication of this is that there is a need to develop and manage a wide range of ‘interfaces’ between the Community of Practice and those outside it. These interfaces could be formal, e.g., in terms of Press conferences or briefings to politicians, or informal, e.g., in terms of reassuring members of the public. Central to these interfaces is the need to define the ‘meaning’ of an incident at the most appropriate level of detail.

The information sources provide frames (Klein et al., 2006a,b, 2007) for interpreting and responding to the incident. Of particular interest are the institutional frames that are designed to aid the management and recording of incidents, such as the electronic forms that allow call handlers to enter information into incident logs. These electronic forms are a repository of prior experience of the organization; they reflect the primary types of incident to which responses are required and the primary types of information that need to be recorded in order to produce consistent, structured accounts of the incident and the response. In addition to these electronic forms, other types of institutional frame are the policies that local police forces might enact, either in response to National policy or in response to local crime patterns. These policies could emphasize the importance of prioritizing response to some types of incident. Finally, institutional frames could come from the collective experience of the personnel involved in incident response, i.e., the community of practice of incident responders, in terms of expectations of how an incident might develop.

The notion that institutional frames can influence decision making echoes the question posed by Manning (1988), viz. “How does organized rationality interface with the variegated dilemmas and perplexities of human communication?” (p. *xv*). Our reading of this question is in terms of the potential conflict between the Naturalistic Decision Making that personnel involved in incident response will apply and the ‘rules’ that are embedded in the forms and procedures that they apply. For Manning (1988), these ‘rules’ might be informal, reflecting concerns of Police Officers, Incident Controllers and Call Handlers (in terms of acceptable ways of behaving on and off duty) and which we see as constituting the community of practice of incident response. Additionally, the ‘rules’ might be formal and dictate how information is recorded, shared and acted upon, i.e., as institutional frames. From the perspective of macrocognition, this patchwork of ‘rules’ will influence the space in which information is interpreted, and the ways in which different ‘framing’ of the same information can vary.

### Incident Response and Macrocognition

Incident response has been extensively researched for major and catastrophic incidents (Dynes, 1970; Quarantelli, 1999; Boin, 2004; Mendonça et al., 2007; Becerra-Fernandez et al., 2008; von Lubitz et al., 2008; McMaster and Baber, 2012). There has been less work on the routine incidents that emergency services face on a day-to-day basis (e.g., Blandford and Wong, 2004, explored Situation Awareness of operators in medical dispatch). Incident response tends to follow a standard process in which a call is received by a Call Handler, responding units are dispatched by the Incident Controller and these units attend and resolve the incident, and the incident in closed. Over the course of this process, an Incident Log is maintained to record relevant information and personnel communicate with each other (via radio) and with members of the public (via telephone or face-to-face).

**FIGURE 1:**
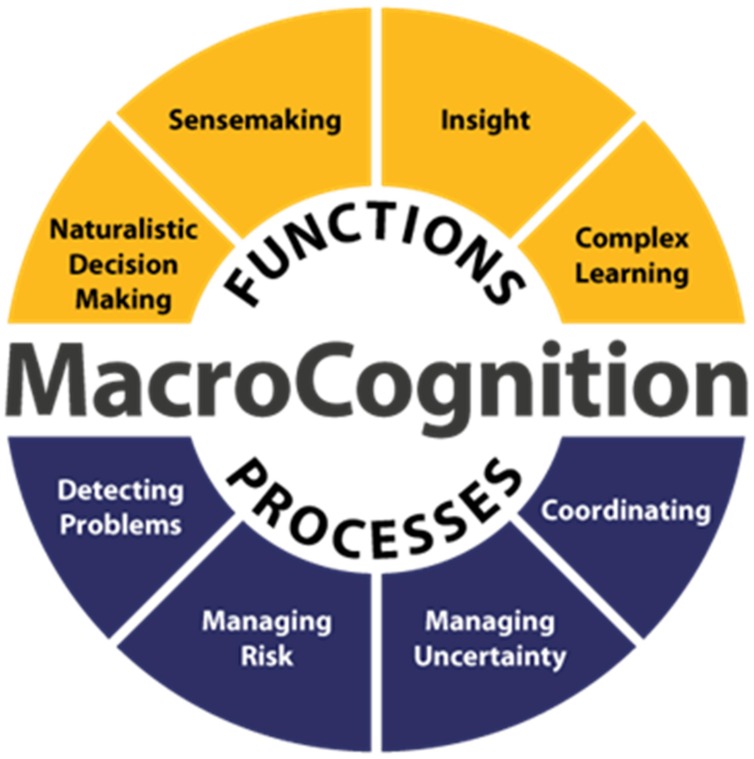
**Processes and functions in macrocognition [from Schraagen et al., 2008]**.

**Figure 1** illustrates core processes and functions related to macrocognition. The processes {detecting problems, managing risk, managing uncertainty, coordinating} are central to incident response. Indeed, these are the primary processes involved in this activity (the only addition here is the process of managing the Incident Log – which we will argue is an essential part of incident response, not only in terms of recording what has been done but also as part of the coordinating process). In terms of the functions, we will present examples of incident response to show how the situations and prior experiences of personnel involved in the response can exhibit characteristics of Naturalistic Decision Making and Sensemaking. We have less to say on Insight and Complex Learning in this paper (although both can play important roles in the response to incidents and handling of crime).

Central to the activity of the Incident Controller is the need to ensure an optimal resource has been dispatched to the incident: too few officers and there might be a risk to the officers or the public, or they might be unable to apprehend the suspect; too many and there could be problems in resourcing subsequent calls. As Blandford and Wong (2004) note, the decisions governing how to resource a response is as much a matter of situation awareness as it is of policy, and the situation awareness includes not only the location and availability of units which could respond but also the type of response which is required.

## Methodology

Over the course of 5 years, the second author worked as a Special Constable (volunteer officer) for a Police Force in the UK. During this time he received training on incident response and attended incidents, working 70 shifts in a two-officer patrol crew deployed in a marked police vehicle. These participant observation sessions enabled direct access to the ‘on the ground’ incident response process, something which is not normally possible for researchers. Informal interviews were conducted with crewmates after incidents had been resolved; notes were taken during patrols whenever possible. These were later supplemented with electronic incident logs for timings and other details. In addition, permission was granted to collect data from the communications centers of two Police forces; over the course of some 30 data collection sessions, we were able to interview and observe Call Handlers and Controllers at work, listen in to 999 (emergency) calls and Police radio traffic, review electronic incident logs. Interviews were done on an opportunistic basis – with questions tailored to clarify the activities that had just been observed. During these interview and observation sessions, data capture was limited to note taking, which ranged from detailed descriptions of activities being undertaken to verbatim recording of telephone and radio conversations.

Such access resulted in a wealth of material. However, this leads to the inevitable problem of deciding what material to select and report. While it is tempting to select those incidents in which there is some level of excitement or novelty, this does not reflect day-to-day operations. On the other hand, some of the more common incidents reveal little of interest about the nature of incident response. For example, a spate of incidents in which gardening equipment was stolen from sheds in back yards might take up a sizeable portion of time but does not make for interesting reading. Typical examples of day-to-day incidents include:

• Burglaries in progress;
• Criminal damage (including arson);
• Domestic violence;
• Medical emergencies (including suicidal and acute mental health problems);
• Retail thefts;
• Road traffic incidents
• Serious assaults;
• Street robberies;
• Urgent welfare concerns (e.g., elderly and disabled persons collapsed in their homes);
• Vehicle crime (e.g., theft from, theft of and driving offenses).

These different types of incident present a range of challenges and risks to the public and responding Officers. Thus, the type of incident will dictate the approaches that are used to respond to them (Flin et al., 2007). For this paper, we have selected a set of incidents that reflect the need for immediate attendance with the opportunity of arresting the suspect (burglaries in progress) or the need to attend the scene to provide assistance (street robbery). We make no claims as to how representative these incidents are of day-to-day policing; we estimate that such incidents would occur three or four times a week, rather than daily, but they represent examples of incidents that those involved would recognize as common. Furthermore, we have chosen not to report incidents which involve violence to the person or domestic violence that contains details which are harrowing and difficult to read.

We present the incidents in two ways. The first is through the use of short vignettes, in which excerpts from incident logs, or verbatim transcripts of radio traffic, are taken from a single incident. The incident transcripts reflect as much information as we feel is necessary for the reader to appreciate what is being discussed or recorded, while also respecting the need to maintain a degree of anonymity in the recorded information. The second is in the form of graphical depictions, which represent the distillation and interpretation of multiple observations and thus are general descriptions of the macrocognitive activities being described. These presentations complement one another, with the vignettes helping the reader to view the diagrams, which in turn provide a framework within which the activity described in the vignettes takes place.

Given the opportunity to collect data in this manner, it is appropriate to ask whether alternative approaches could have been feasible or produced more reliable data. We opted for a participant observation and interview-based approach, with the primary focus on the Police officers and associated staff and the processes that they follow. This means that, in comparison with an ethnographic approach (Hammersley and Atkinson, 1995), this study is heavily prescribed by the information flow and operating procedures. Our descriptions show how information is received, processed and passed around the system. What we are not capturing in detail are the assumptions, attitudes and expectations of the personnel involved (or, for that matter, the members of the public who are the subject of these processes). Thus, while the examples used in the paper involve researchers participating in the social practices under investigation, the process-oriented analysis could miss the rationalization through which the participants continually revise their understanding of the situations they encounter. In other words, we are taking the behavior of participants as indicative of the processes that they follow and then inferring the ‘meaning’ that these processes involve. Where practicable we have sought to corroborate our interpretation of meaning with the participants, but the study is not focused on extracting notions of sense and meaning directly from the participants. We believe that the approach taken provides opportunity to triangulate data (through multiple sources of information being collected for each example), investigator (through continued exploration of assumptions made by the two authors in their analysis) and theory (through developing an explanation of the processes that we are observing).

### Coding, Synthesizing, and Representing the Data

For the textual descriptions of the incident, the presentation format is to use CAPITALS to indicate material typed in to the Incident Log (with time of entry on the left), i.e.,

**14:20** Controller 1: “*THE IP HAS BEEN STRUCK AND FELL TO THE FLOOR*”

and for verbal communications to be presented in *italics*, i.e.,

Whiskey 3-5: “*Yes – confirmed break-in.*”

In the examples in this paper, the textual descriptions are verbatim accounts recorded *in vivo*.

The graphical description was originally developed in McMaster and Baber (2005) and is intended to show how cognitive activity in spread across actors and artifacts. **Table 1** lists features of the activity and which can be used as the basis of a simple task analysis.

**Table 1:** Features of Activity.

Feature	Description
Agents	Who is involved (people/artifacts)
Activity	The purpose of the operation
Modality	Information state (verbal, text, etc.)
Form	Language style, abbreviations, etc.
Transmission	How is information shared
Transformation	How is information acted upon
Storage	How is information retained
Resource for action	Actions cued by representation

**FIGURE 2:**
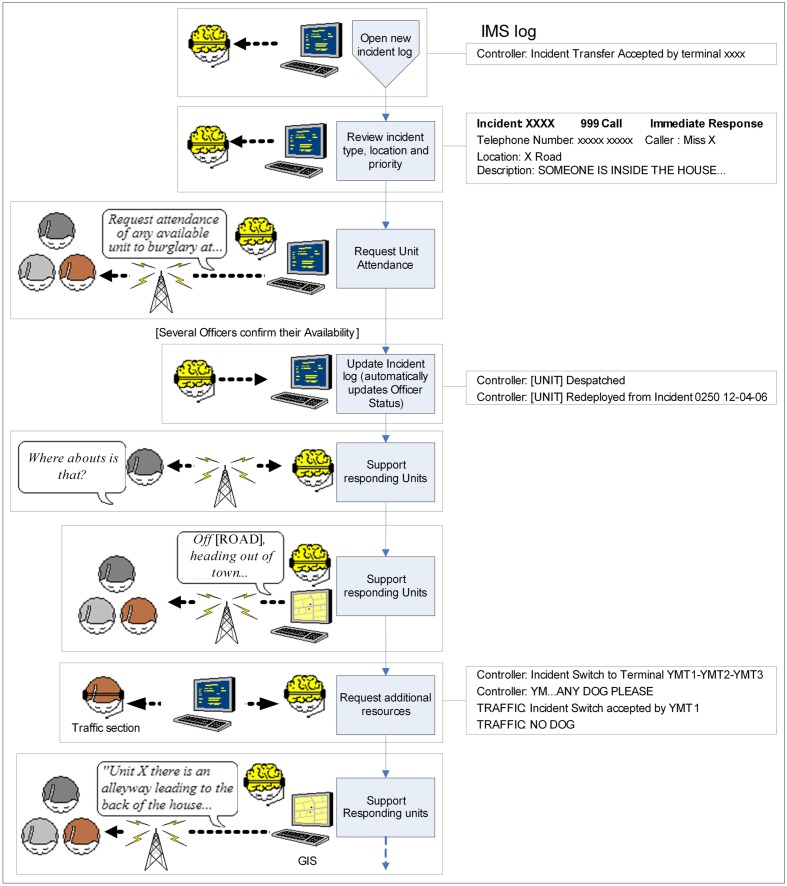
**Negotiating meaning: determining the location of an incident**.

The features from **Table 1** are combined into a diagram which shows the flow of information in an incident response (**Figure 2**). The diagram shows the key transformations of information (e.g., from one modality or storage medium to another). Thus, **Figure 2** shows the process through which an Incident Controller (in the first panel) responds to an open Incident Management System (IMS) log for an incident requiring immediate attendance, and then puts out a call to all units to ask for attendance. Of the units that respond, one unit asks for further details on the location. As the incident unfolds, the Incident Controller provides further information relating to access to the property.

While **Figure 2** provides a summary of the incident, we are aware that such a representation is not without its problems. Any description (verbal or graphical) stands or falls on the comprehensiveness of its content and, consequently, reflects the selectivity of the analyst. As far as practicable, we have included those elements of the incident which were ‘external’, i.e., available to participants in the incident response, e.g., the content of the Incident Log, verbatim transcriptions of communications over the radio. This means that we have not included the reflections, assumptions, interpretations and other ‘internal’ elements of the responders. Nor have we provided much in the way of contextual or situational material for each incident. However, we feel that the material that we report is sufficient to allow us to draw conclusions relating to the macrocognition involved in incident response.

The approach to coding of the examples, in terms of type of meaning, is explained for each example. In broad terms, where participants are asking questions or where there is evidence of confusion, we consider this to be negotiated meaning. Here, the participants are, we believe, seeking to establish common ground in order to make sense of the incident. Where participants are giving direct instructions, we consider this to be actionable meaning. Here, the participants are either providing information or instructions that enable other participants to effect an action. Where information is being typed in to the Incident Log, we consider this to be structured meaning. Here, the information is being formatted for subsequent use. As the examples illustrate, this distinction is not always clear-cut; information might be structured (in the sense that it is typed in to the Incident Log) but could also involve negotiation, with participants raising questions or debating the meaning of the information. We also note that several of the examples show overlap in the types of meaning, i.e., it is rarely the case that the incident proceeds with negotiated meaning at the start, leading to actionable meaning and then to structured meaning in the final report. Rather, the incidents appear to shift between these meaning types.

## The 999 Call: From Negotiated to Actionable Meaning

Whalen and Zimmerman (1990) show Callers often present imprecise and hesitant openings to their calls. As Baber et al. (2006) point out, rather than taking a verbatim account of the Caller’s information, the Incident Controller will translate this information into a format which is more suited to the structure of the Incident Log. Thus, one of the roles of the Call Handler is to negotiate the meaning of the incident with the Caller. This negotiation is supported by a set of core questions that Call Handlers are trained to use in order to direct the conversation and to establish the important facts quickly, e.g.,

• Call Handler: “*Have you been injured?*”
• Call Handler: “*Where did they go?*”

Caller, Call Handler, and Incident Controller develop some form of common ground (Clarke, 1996). In this concept, common ground is “*the mutual knowledge, beliefs, and assumptions shared by the speaker and addressees.*” (Clarke, 1996, p. 247). Clarke’s (1996) concept of common ground proposes that people draw on three sources of information:

• Perceptual evidence (the experience to which people have access);
• Linguistic evidence (the words that people are hearing);
• Community evidence (knowledge which they might believe is shared within a given community, perhaps as the result of training or enculturation).

The Caller, Call Handler and Incident, Controller will not have the same perceptual evidence (the Call Handler and Incident Controller are removed from the scene that the Caller is witnessing or recalling). Thus, part of the conversation is aimed at translating the Caller’s perceptual evidence into actionable meaning (to support the Incident Controller) and part of the conversation is aimed at translating the Caller’s perceptual evidence into structured meaning (to support completion of the Incident Log). In terms of linguistic evidence, a key role of the Incident Controller is to translate the words of the Caller into the terminology used by the Police. For instance, the description of an offender may change from “*white lad*” to “*IC1 male*”, which is the relevant UK Police National Computer Ethnicity Classification. Abbreviations and acronyms are also employed, for example “*My car has been stolen*” is formalized within the Police as “*Theft of Motor Vehicle*”, which is written as “*TOMV*”. This terminology and jargon relates to community evidence. Furthermore, one might find Caller’s seeking to provide information in a manner which they believe fits the community knowledge of the Police, e.g., when reporting a car’s registration number, the Caller might use the ICAO (International Civil Aviation Organization) alphabet of A, alpha; B, bravo; C, Charlie etc. because they believe that this is how to report letters of the alphabet to a Police Officer. Of course, the Caller’s might not know all of the words used in the ICAO alphabet and so might use their idiosyncratic versions, such as A, apple; B, baby etc., but the intent of providing clear definition of letters over a potentially noisy communication channel remains the same.

For us, common ground represents the meaning that the Caller and Call Handler are negotiating during the call, and which is then translated into structured meaning by the Call Handler to record onto the IMS so that it could be read by an Incident Controller, interpreted in terms of actionable meaning, and subsequently communicated over the radio to responding units. In terms of macrocognition, ‘common ground’ implies the need for a community of practice to work within its institutional frames to gather the appropriate ‘community evidence’, i.e., that information is selected which corresponds to working practices and which has been recorded in an acceptable. The processes by which information is selected and recorded relate to our notions of ‘meaning’. **Figure 2** illustrates some of the issues surrounding common ground in incident management. In response to an initial call, the Incident Controller issues a ‘Request for attendance’ to the incident at ‘x road’. The first response to this request is to ask ‘where abouts is that?’ to which the Incident Controller provides further geographical information. Here, the relevant information is being explored and elaborated in a form of negotiated meaning. As Attending Officers reach the address, the Incident Controller provides further information about the geography (‘…an alleyway leading to the back of the house…’). In this example, the unfolding activity can be seen as the effort after actionable meaning, i.e., to provide sufficient information to the Attending Officers to allow them to operate at that address.

The incident summarized in **Figure 2** has an Incident Log entry of “no dog”, indicating that it is not possible to supply a police dog to this call. In order to make these decisions, the Incident Controller draws on the incident classification made by the Call Handler in response to the original call and recorded the Incident Log. Often the classification (and required response) is negotiated through the editing of the Incident Log as the response unfolds. What we find particularly interesting is the decision of what to include in the Incident Log; when the Incident Controller (and Call Handler) speaks to Attending Officers, members of other services or members of the public, what is recorded is not a verbatim account but as accurate a gist of the conversation as is sufficient for the log. At this level, macrocognition applies to the translation of negotiated meaning (i.e., the content of conversations which might require clarification) into structured meaning (i.e., which can be written into the Incident Log).

**FIGURE 3:**
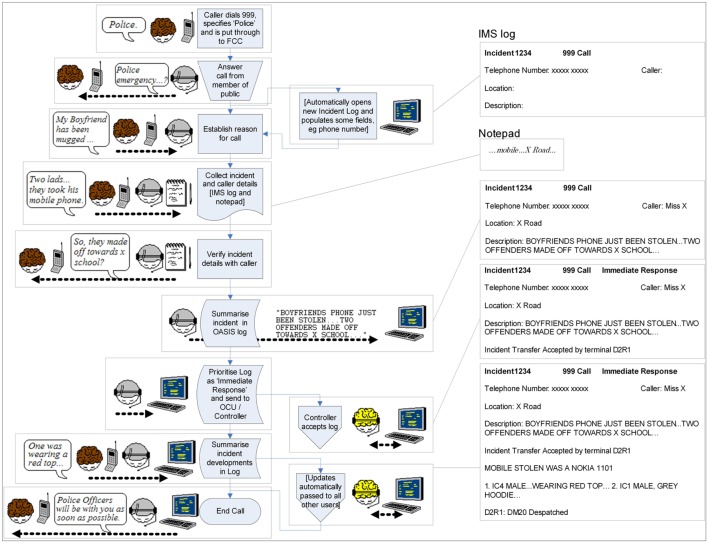
**Actionable meaning: determining the nature of a response**.

**Figure 3** summarizes the process of taking a 999 call arising from a street robbery. The boxes on the right-hand side of the figure show the information that is recorded in the notepad and incident log at various points, showing how the incident log gradually develops during the course of the call. The figure also illustrates how the log structures the incident details and mediates indirect communications between the Call Handler and Control (the Call Handler can see that Control has dispatched a unit to the incident and is able to tell the Caller that the Police will be with them soon).

In the incident summarized in **Figure 3**, the caller provides initial information about the incident, i.e., “My boyfriend has been mugged…Two lads…they took his mobile phone.” While this provides some information about the nature of the incident, it does not provide information that might be relevant to the response, such as whether any injuries had been sustained. Thus, the initial call log records a location and a likely destination for the perpetrators, i.e., ‘x school’. Again, the aim is to provide sufficient actionable meaning for the response to be made.

In the following extract, two Incident Controllers are jointly handling multiple incidents on the same radio talk group; they update the same Incident Log relating to the ongoing reporting of a violent robbery. The timestamps (minutes and seconds since the start of the call, on the left of the text) indicate when information is typed into the log; where there are gaps in the timestamps, e.g., 14:27 to 14:58, this is likely to be where one of the Controllers is speaking with the Officer Attending. In this log, two issues are raised and resolved. The first issue involves concerns with the victim Injured Party (IP) of an attack and whether or not an ambulance (Ambo) is needed. The second concerns the need for Scene of Crime Officers (SOCO) to attend the scene to gather evidence (which involves notifying a third controller).

**14:20** Controller 1: “*THE IP HAS BEEN STRUCK AND FELL TO THE FLOOR*”
**14:27** Controller 1: “*OFFICERS CHECKING TO ASCERTAIN IF AMBO REQUIRED.*”
**14:58** Controller 2: “*LADY HAS BEEN KNOCKED OVER AT DOOR WHEN OFFENDERS*”
**15:00** Controller 2: “*GAINED ENTRY*”
**15:15** Controller 1: “*CAN SOCO ATTEND ASAP PLSE*”
**15:30** Controller 1: “*FROM OFFICERS THE FEMALE IP DOES NOT REQUIRE AMBO AS*”
**15:32** Controller 2: “*PLS GET SOCO FOR THIS*”
**15:39** Controller 1: “*IP STATES HAS NO INJURIES*”
**16:06** Controller 2: “*ASKING FOR AMBO ELDERLY FEEMAL BADLY SHAKEN APPROX*”
**16:07** Controller 2: “*86 YRS*”
**16:23** Controller 3: Incident Accepted
**17:36** Controller 1: “THE OFFICERS NOW ASKING FOR AMBO AS THE IP 86YRS OLD”
**17:51** Controller 1: “IS EXTREMELY DISTRESSED-UPSET”
**17:54** Controller 2: “AMBO LOG [Number]”
**18:34** Controller 3: “SOCO INFORMED.”
**18:40** Controller 3: This incident added to SOCO list for section [Number]

It is noteworthy that in this example there is no spoken communication between the three Incident Controllers, two of whom are co-located. Rather, the updating the Incident Log provides the development of common ground concerning the incident. Thus, Controller 1 (15:30) suggests that an ambulance is not required but subsequently Controller 2 (16:06) disagrees and requests an ambulance. Both entries are made in response to comments from the Attending Officers (as indicated in the Incident Log) and, so the change in entries reflects changes in the assessment of the situation made at the scene. For Controller 1, there was no need for the ambulance as the Attending Officers report that the IP “has no injuries” (15:39) but for Controller 2, they note the age of the IP and the she is “badly shaken” (16:06). Controller 1 then also logs the request for ambulance as the IP is “extremely distressed – upset” (17:51). Controller 2 logs the request for an ambulance to attend (17:54). In this example, the updating of the Incident Log provides both a record of the management of the incident, i.e., structured meaning, that could provide the basis for subsequent enquiries (i.e., the condition of the victim could be used as part of the prosecution against the perpetrator) and negotiated meaning, i.e., in terms of deciding whether or not to call for an ambulance. The example concludes with actionable meaning, i.e., an ambulance is called and a Scene of Crimes Officers is tasked with visiting the scene. What is particularly pertinent about this example is that way in which the three types of meaning are interspersed, and the way in which the negotiation is performed through comments on the Incident Log rather than through verbal communication (even, as we have already noted, two of the Incident Controllers are adjacent to each other in the control room).

## Attending Officers Traveling to the Incident

As they make their way to the incident, Officers plan their response in terms of risk (threat assessment), powers and policy, and tactics. For Borglund and Nuldén (2008) this represents a form of ‘active traveling’, in which the Officers will not only search the streets at they drive for vehicles or persons of interest and for the address to which they have been directed, but also review experience of previous, similar calls. Although the Officers will have received some initial details from the Controller, these are often only the bare minimum, such as an approximate location and a statement of the nature of the incident, for example “*male being assaulted by two males*”. In terms of the macrocognitive process of ‘managing risk’ (**Figure 1**), the first indication of the level of risk associated with the incident (both to members of the public and the responding Officers) and consequently the appropriate response, will come from the type of incident. When an offender is named by the Caller, Officers might ask the Controller to run a check through the Police National Computer; if the person is known to the police, this will provide a summary of any previous arrests or convictions, as well as warning markers (i.e., drugs, violence, weapons or self-harm) associated with those individuals.

If the Call Handler updates the log as a result of further conversation with the Caller (e.g., description of an offender, their direction of travel, vehicle, etc.), this information will be visible to the Controller, who passes this to the Officers. As a result of these further updates, the responding units may change their tactics, for example, if the offender has left the scene Officers may decide to perform a search of the area before speaking to the victim, in the hope of catching the offender.

In terms of the macrocognitive processes of ‘managing uncertainty’ and ‘detecting problems’ (**Figure 1**), Attending Officers may ask the Controller to check IMS for: previous emergency calls to that location, details of any persons associated with that location and any previous convictions or warning markers (e.g., for violence or weapons) associated with those individuals. For example, the IMS will indicate if previous 999 calls have been made from a number, or if any persons named in a log are associated with previous incidents at that address. In their analysis of Mobile Data Terminal (MDT) use, Branaghan et al. (2010) identified five main clusters of information which could inform decision making of Attending Officers: Potentially Violent, Citizen Welfare, Medical, Traffic, Non-violent. These relate to the macrocognitive demands related to managing uncertainty, managing risk and detecting problems, and could the subject of discussion between Attending Officers and Control, or amongst Officers in a talk group.

Officers will often rely on Controllers to remind them of incident details that they have forgotten – such as house numbers, names, or vehicle registration numbers – radioing the Controller as they near the scene to request that that information is repeated. In **Figure 2**, an Officer asks for some clarification of where the incident location was, and then asks for the name of the company to be repeated. The Controller has pro-actively checked the location using the GIS (Geographical Information System) and, unprompted, provides information to clarify the incident location, i.e., in terms of the ‘alleyway’ to the back of the house.

## Attending Officers at the Scene

As they arrive at the scene, responding Officers notify the Controller (who updates the incident log); the Officers may be confronted by an ongoing incident, or they may find that the immediate threat from the incident has stopped. Their response to the incident is concerned with: (i) controlling and resolving the situation, and (ii) performing an initial investigation of the events surrounding the incident. Where more than one Officer is deployed to an incident, they may decide to separate and divide tasks between them (e.g., conducting searches, separating belligerent parties, speaking to witnesses), using their radios in point to point mode (i.e., direct one to one) to coordinate their activities without taking up airtime on the talk group.

In terms of the macrocognitive process of ‘managing uncertainty’ (**Figure 1**), responding to incidents is complicated by the fact that many of the incident details may well be inaccurate, including the caller’s account of events, the names or descriptions of parties involved and very often the nature of the incident itself (i.e., the frame selected by the Call Handler during the initial call). In the following example, multiple units respond to reports of a break-in in progress at night; Officers are on the scene within 3 min, however, on their arrival, the property and surrounding houses appear to be secure and undisturbed, casting doubt on the nature of the incident. The Controller switches the incident log back to the Call Hander (in a different Control Room) to double check the address. The situation Officers encounter at the scene is at variance to the summary they have been given, which, in turn, cues activity from the Controllers and Call Handler, who communicate with each other via the IMS (12:46 to 13:28).

**12:46** Controller A: “*CAN YOU CONFIRM x RD OR x ST*”
**13:00** Call Handler: “STANDBY”
**13:23** Call Handler: “*I HAVE LISTEND TO TAPE AGAIN IT IS x STREET*”
**13:28** Call Handler: “*NOT ROAD - MY APOLOGIES*”
**13:28** Controller A: [Receives no reply from caller’s mobile phone.]
**14:10** Controller B: [Updates caller details to x Street]
**14:16** Controller A: [Updates incident location to x Street]
**15:20** Controller B: [Notes that the house numbers in x Street only go up to 12 – the caller had reported living at number 15]
**15:50** Controller B: [Performs searches for the caller on the Electoral Role database]
**17:20** Call Handler: “*I HAVE LISTEND TO ALL THE TAPE AND WHEN I CONFIRM*”
**17:34** Call Handler: “*THE NUMBER OF THE ADDRESS CALLER STATES x ROAD*”
**17:44** Call Handler: “*I REPEATED IT TO HIM AND HE SAID YES x ROAD*”
**17:54** Call Handler: “*AT THE BEGINNING OF THE TAPE HE STATES x*”
**18:06** Call Handler: “*STREET*”
**21:50** Controller B: [Notes that Officers have checked the front and rear of both 12 x St and 12 x Rd and spoken to resident at 12 x St – all in order.]
**24:50** Controller B: [All units are leaving the scene. The log is closed, having been redefined as a false call.]

While we have presented the types of meaning as related to common ground, this does not guarantee that all communications are correct or complete. The notion of negotiated meaning that we are developing in this paper suggests that it is possible for a community of practice to carry more than one interpretation of a situation. These multiple interpretations could arise from problems with the structured information, e.g., in the previous example, the problem was whether the address was ‘road’ or ‘street’. As soon as it became apparent that the incident could not be resolved, it was closed as a ‘false call’. In this example, the ‘common ground’ was not necessarily agreement on the address so much as agreement on the nature of the call (and how to respond to it).

In exceptional circumstances, the talk group becomes an open forum for a group of responding Officers to collaboratively make sense of an incident. This extract shows part of the radio communications during the response to a ‘break-in in progress’ (burglary), where several Officers were already at the scene, searching for the offender and other resources were en route. As can be seen, Officers are using the talk group to directly communicate in order to coordinate their response, with the Controller playing an ancillary, rather than leading role. Interestingly, although the Sergeant involved provides some leadership to the other units – for example directing units during the search – none of the units involved in the example is demonstrably ‘in charge’ of coordinating the response. Instead, the units involved jointly make sense of and determine the response to the incident (break-in in progress) and the situation as they find it. This also shows that the Controller has to repeat the incident details several times, either because a new unit has become involved (Dog Handler), or because details have been forgotten (Whiskey 2).

W3-5[Sergeant]: “[OFFICER A]*: you’re on the wrong side….Unit looking at me, go down there.*”
Dog Handler: “*You were calling me?*”
Control: “*Possible Break in progress…*” [Gives details]
Dog Handler: “*Can you confirm I’m required?*”
Whiskey 3-5: “*Yes – confirmed break-in.*”
…
Whiskey 2: “*Whiskey 2: What’s the address again?*”
Control: “[ADDRESS]”
[Confusion ensues over the location of the road and property]
Control: “*On mapping, you have got* [ROAD]*…*”
Officer A: “*I’m by* [LOCATION]*, is that right?*”
Officer B: “*No, it’s further round, near the church….do a left there.*”
Officer C: “[OFFICER C] *to 3-5.*”
Whiskey 3-5: “*Go on.*”
Officer C: “*Can you speak to the IP and see if a laptop’s been stolen?*”
Whiskey 3-5: “*Confirmed.*”
Officer C: “*I’ve found a laptop cable…*”
Whiskey 3-5: “*Does that give a direction of travel?*”
Officer C: “*It goes to a dead end…*”
Whiskey 1: “*Whiskey 1 to Control?*”
Control: “*Go ahead.*”
Whiskey 1: “*Another property is open*, [OFFENDER] *may still be inside.*”
Whiskey 3-5: “[Requests location of this address]”
Whiskey 1: “*…outside IP’s address, go back…2nd right…*”

In this example, the different threads of conversation show interconnections between different types of meaning. The negotiated meaning develops over the course of response, e.g., in terms of tasking (‘can you confirm I’m required?’, ‘see if a laptop’s been stolen?’) and in terms of location (‘what’s the address gain?’, ‘is that right?’, ‘it goes to a dead end…’). Incident Controller (Control) is providing information to Attending Officers, in the form of the specific location of the incident. The Attending Officers are sharing information with Control (‘another property is open [Offender] may still be inside’). This example captures some of the confusion of incident response, with the need to define the required information to support the response, and the manner in which response can develop as new opportunities arise. The multi-threading of meaning in this example shows how the *ad hoc* planning of incident response creates opportunities to develop common ground between the community of practice. It also provides an interesting insight into the challenges of defining what information to record in the Incident Log, i.e., when to convert the information to structured meaning.

## Closing the Incident

Once the incident has been resolved, the Officer will radio the Controller with a final update that summarizes their assessment of the incident and the actions taken. This narrative could be as short as “*One under arrest for drunk and disorderly – transporting to Custody”*, but may be more lengthy for complex incidents. The Controller will add this final update to the incident log, which is then closed.

## Discussion

We began this paper with the proposal that sensemaking, as collaborative activity, is performed within a given community of practice, operating with the constraints of its institutional frames (which are both formal and informal rules of that community and the technology used to support its activity). In the examples presented in the paper, the rules are instantiated through the ways in which the community of practice manages meaning. As the examples show, the management of meaning is not a neat, linear process but involves the participants raising questions, seeking clarification, misinterpreting information and correcting their understanding. We have used the notion of common ground as a lens through which to consider this activity, but it is also illustrates very nicely the cyclical nature of sensemaking in the Data/Frame model (Klein et al., 2006a,b).

In the examples, the manner in which information is communicated influences the ways in which meaning is managed. For the Attending Officers, communication is almost exclusively spoken, either via radio or face-to-face. This means that Attending Officers tend to only know the content of Incident Log when it is read to them by the Incident Controller. In this case, macrocognition applies to the translation from structured meaning to actionable meaning (i.e., from the entries in the Incident Log to advice and instruction for Attending Officers). As Clarke’s (1996) notion of common ground implies, the macrocognition of incident response is a continual process of comparing and contrasting across perceptual, linguistic, community evidence. From another perspective, the notion of Distributed Situation Awareness (Stanton et al., 2006) suggests that teams will typically have different views on a situation, with the possibility that their knowledge overlaps in part rather than completely. This suggests that the macrocognition in incident response relates to deciding what knowledge to share and what format to use for its sharing. For example, the response to the “intruder” shining a torch into someone’s window involved sharing of knowledge of previous incidents from this address. This ‘informal’ knowledge could be shared during the briefing prior to patrols leaving the Police Station or could, as in this instance, be shared over the radio. In this instance, the shared knowledge became integral to the response, i.e., “we’ll go and have a chat”. When this knowledge applies to the response, it is formally recorded in the Incident Log. Otherwise, it remains part of the informal ‘rules’ that play against the formal rules for recording. The examples also highlight that the interplay of formal and informal is not a simple matter of all entries in the Incident Log having structured meaning, i.e., there are several examples in which the content of the Incident Log is used to challenge other entries; in such cases, the ‘formal’ (structured meaning) of the Incident Log is replaced with an ‘informal’ (negotiated meaning). What is interesting here is that such communication can occur even when a more appropriate channel for informal communication is available, i.e., when Incident Controllers are sitting near each other and can simply talk to each other. This suggests that the notions of formal/informal rules, or negotiated/structured meaning are neither rigid concepts to apply to analysis nor necessarily factors consider in the choices that Incident Controllers make.

In terms of limitations of the work, the use of a selection of examples taken from a larger collection could raise accusations of ‘cherry-picking’ those examples which best support the points that we are making in the paper. It might have been beneficial to report more examples, or to classify a large collection of examples in terms of the issues identified in the paper. We feel that the examples illustrate the individual nature of the incidents that Police will be responding to. This means that collecting more examples might not necessarily allow reduction to specific types, and hence there is a need to consider individual cases. On the other hand, in order to determine whether the unique characteristics of a specific case can be generalized to similar operations, there is a need to extend the set of examples that are explored, and this could be the subject of subsequent work.

In terms of the lessons that these examples, and our analysis of them, might raise, we believe that there are two lines of exploration that could be developed. The first concerns the nature of sensemaking as collaborative activity. Many of the case studies that have been reported since Weick’s (1995) pioneering work on sensemaking draw on analyses of discussions and meetings in which groups make sense of the problems that they face. Thus, there seems a strong case to be made for the proposal that collaborative sensemaking follows the elements outlined in this paper. However, the idea that there is an ‘informal’ sense which can be used to describe and define a situation only covers part of the processes that sensemaking involves. For many situations (and this is often critical in Emergency Response) there is a parallel requirement to produce a ‘formal’ statement of the response and this requires description of the situation in terms which can be used to justify the use of resources. Baber et al. (2006) describe how narratives are constructed to develop the crime scene investigation from informal sensemaking to formal reporting. The Incident Log, which is a formalized ‘in the moment’ account of the incident response as a series of time-stamped event updates which reflect the twists and turns of the ongoing sensemaking process that took place during the incident.

The second line of exploration concerns that nature of the technology and work processes followed in Incident Response. As Manning (1988) notes, there is an ongoing tension between the need to record a formal, reliable, and objective account of the response, and the collaborative search after meaning, which seems to arise spontaneously when groups of people engage in sensemaking. One implication of this is the need to manage the ‘meaning’ of the incident as it unfolds, and to combine this with the management of the incident itself. For us, this implies two cycles of macrocognition which partially overlap. The first cycle concerns the formal rules which govern the management of the response, e.g., in terms of recording details in the Incident Log and in terms of providing resources for the response. The second concerns the informal rules which govern the operation of the Community of Practice and support the managing of uncertainty and risk as the incident unfolds. It is this overlap between these two cycles of macrocognition which enables adaptability in the ensuing response and which also the need to ensure that the ‘formal’ rules do not overwhelm the informal rules.

## Author Contributions

The paper was written by CB. The examples and diagrams were collected by RM. Analysis and discussion was written by CB.

## Conflict of Interest Statement

The authors declare that the research was conducted in the absence of any commercial or financial relationships that could be construed as a potential conflict of interest.
